# Measuring and paying for quality of care in performance-based financing: Experience from seven low and middle-income countries (Democratic Republic of Congo, Kyrgyzstan, Malawi, Mozambique, Nigeria, Senegal and Zambia)

**DOI:** 10.7189/jogh.08.021003

**Published:** 2018-12

**Authors:** Jessica Gergen, Erik Josephson, Christina Vernon, Samantha Ski, Sara Riese, Sebastian Bauhoff, Supriya Madhavan

**Affiliations:** 1 Visualst, Maputo, Mozambique; 2 Independent consultant, Washington, D.C., USA; 3 Independent consultant, Bethesda, Maryland, USA; 4 University Research Co., LLC (URC), Chevy Chase, Maryland, USA; 5 Center for Global Development (CGD), Washington, D.C., USA; 6 United States Agency for International Development (USAID), Washington, D.C., USA

## Abstract

**Background:**

Performance-based financing (PBF) both measures and determines payments based on the quality of care delivered and is emerging as a potential tool to improve quality.

**Methods:**

Comparative case study methodology was used to analyze common challenges and lessons learned in quality of care across seven PBF programs (Democratic Republic of Congo, Kyrgyzstan, Malawi, Mozambique, Nigeria, Senegal and Zambia). The eight case studies, across seven PBF programs, compared were commissioned by the USAID-funded Translating Research into Action (TRAction) project (n = 4), USAID’s Health Finance and Government project (n = 3), and from the Global Delivery Initiative (n = 1).

**Results:**

The programs show similar design features to assess quality, but significant heterogeneity in their application. The seven programs included 18 unique quality checklists, containing over 1400 quality of care indicators, with an average per checklist of 116 indicators (ranging from 26-228). The quality checklists share a focus on structural components of quality (representing 80% of indicators on average, ranging from 38%-91%). Process indicators constituted an average of 20% across all checklists (ranging from 8.4% to 61.5%), with the majority measuring the correct application of care protocols for MCH services including child immunization. The sample included only one example of an outcome indicator from Kyrgyzstan. Performance data demonstrated a modest upward improvement over time in checklist scores across schemes, however, achievements plateaued at 60%-70%, with small or rural clinics reporting difficulty achieving payment thresholds due to limited resources and poor infrastructure. Payment allocations (distribution) and thresholds (for payments), data transparency, and approaches to measuring (verification) of quality differ across schemes.

**Conclusions:**

Similarities exist in the processes that govern the design of PBF mechanisms, yet substantial heterogeneity in the experiences of implementing quality of care components in PBF programs are evident. This comparison suggests tailoring further the quality component of PBF programs to local and country contexts, and a need to better understand how quality is measured in practice. The growing operational experiences with PBF programs in different settings offer opportunities to learn from best practices, improve ongoing and future programs, and inform research to alleviate current challenges.

In many resource-poor settings health care services perform poorly across the key domains that often define quality – safe, effective, timely, efficient, equitable, and people-centered [1,2] – and are generally highly variable across patients and providers [3,4]. However, there is limited guidance on what policies can systematically improve quality of care and are financially sustainable and administratively viable [5-7].

Performance-based financing (PBF) is a strategic purchasing approach that offers providers and health facilities a per-unit payment for targeted tasks or service outcomes [8,9]. Quality of care is a central component of many PBF schemes because of concerns that providers may compromise quality when increasing the volume of services in response to the payment incentives [6,10-12]. Moreover, improving quality of care may attract more patients and thereby help achieve desired increases in utilization [6,13]. As a financing approach, PBF is fundamentally different from input-based financing and has generated substantial interest [12,14]. A dedicated fund at the World Bank, the Health Results Innovation Trust Fund (HRITF), has encouraged many countries to consider PBF programs and conducts significant research on these schemes. As of 2015, the HRITF supported 36 PBF programs that focus on maternal and child health (MCH), associated with US$ 400 million in grants and US$ 2.2 billion in concessional loans [15].

Substantial heterogeneity exists in how quality is incorporated in the design and implementation of PBF [6,16]. Differences in design features include the specific set of quality indicators and how they are considered in the payment formula. For instance, particular indicators can be directly rewarded (akin to how quantity measures are generally incorporated) or used to indirectly modify quantity bonus payments, inflating or deflating the bonus according to aggregate quality performance [17]. Similarly, programs vary in their approaches to implementation, with regards to verification, the frequency of payments and how the facilities can use the bonus payments. Preliminary findings from the initial set of rigorous impact evaluations associated with the World Bank’s HRITF suggest that these differences may have varying effects on the potential of PBF to improve the quantity and quality of care [18].

The goal of this paper is to systematically document experiences in measuring and paying for quality in donor-supported PBF programs for maternal and child health (MCH) services across seven low- and middle-income countries (LMICs). Our goal is to provide insights into how the quality of care is implemented and formally documented across contexts, designs, and funders. Our sample of PBF schemes differs in their context and approach to PBF, as well as in their source of external financial assistance. We identify similarities and differences across programs, focusing on various key design and implementation aspects, such as the content of the quality of care checklists, reporting and verification, and how quality is featured in the payment formula.

## METHODS

We used a comparative case study methodology by conducting a desktop review, hosting key informant interviews, and assessing quality performance data. We analyzed design elements, common challenges, and lessons learned in quality of care across eight case studies of PBF programs (Democratic Republic of Congo, Kyrgyzstan, Malawi, Mozambique, Nigeria, Senegal and Zambia).

### Selection/sampling

The case studies on the seven PBF programs come from three sources. Five case studies (Democratic Republic of Congo (funded by USAID), Mozambique (CDC), Nigeria (World Bank), and Senegal (World Bank)) were conducted by this study’s authors and commissioned by the USAID-funded Translating Research into Action (TRAction) Project [19,20]. Three case studies (Kyrgyzstan, Nigeria, and Zambia) were drawn from a recently published report by USAID’s Health Finance and Government (HFG) project [21]. One case study (Malawi) conducted by the Global Delivery Initiative (GDI) [22]. We primarily drew on the TRAction-supported cases and incorporated insights from the other cases where possible. We analyzed the Nigeria PBF program using the TRAction and the HFG case studies and analyzed the Malawi PBF program utilizing the TRAction and GDI case studies.

The five TRAction-supported cases were purposively selected to achieve a distribution of countries that included: USAID Ending Preventable Child and Maternal Deaths (EPCMD) priority countries whose PBF programs focused on MCH health; variation in the source of PBF funding; variation in the extent to which quality measures were integrated into PBF programs; sufficient availability of program documentation and data on quality of care; and sufficient project duration to be able to measure quality changes.

The HFG project's set of three case studies, two of which also cover USAID EPCMD priority countries (Nigeria and Zambia), were selected because the documentation of PBF payment design, implementation duration, and lessons related to maternal health service delivery quality were sufficient to enable robust comparison with the other set of studies [21].

The Malawi GDI case study was included as it aligned with our selection criteria, in that Malawi is also an EPCMD country, the PBF implementation featured is financed by a unique funder relative to the other cases (*Kreditenstalt fur Wiederaufbau*, KfW; and the Norwegian Agency for Development Cooperation, NORAD), and the documentation of program design and outcome elements was sufficient to support its inclusion in our analyses.

### Data collection (completed by case study authors)

Data collection for each of the five TRAction-commissioned case studies began with a review of publicly available materials (program reports, evaluations, manuals, standard operating procedures, and quality assessment tools), followed by qualitative interviews with two to five informants including representatives from the Ministry of Health, donors/funders, PBF implementers, field support staff, and health providers. Key informants were selected using both purposive and snowball sampling. An initial literature review of quality of care in PBF informed the development of qualitative key informant interview guides with input from experts and stakeholders (ie, University Research Co., LLC (URC), USAID). The guide was refined for use in each country adapting to context, PBF project, and specific stakeholder's area of expertise and job function. For instance, field support staff were questioned about the implementation challenges of the quality tool and the impact on facilities, whereas PBF designers and donors/funders were asked about tool design and impact on quality scores.

Data were collected between September 2014 and March 2015 for TRAction case studies, and September-November 2016 for HFG case studies. The GDI case study examines the ongoing Maternal and Neonatal Health (RBF4MNH) in Malawi (project duration: August 2011-December 2017), and includes information from 2010-2015 [17,23]. We also used data on the quality of care performance for the PBF programs in Nigeria, Mozambique, DRC, Kyrgyzstan, and Senegal was shared with our team or publicly available. These data consist of the quarterly quality scores as constructed from the program-specific checklists. We calculated the minimum, maximum and median of checklist scores across the smallest geographic unit (health zones, districts, etc.) per country, as well as a linear trend line over time. We also used the timeframe where no checklist revisions were incurred, so the years reported varying between countries. Information on the quality indicators used in these PBF programs comes from a previously published database of quality checklists that also classifies indicators as capturing structure, process or outcome quality. The database contained multiple entries for Senegal, Nigeria, and Zambia that allow us to describe revisions to the checklists over time (http://www.harpnet.org/resource/multi-country-performance-based-incentives-quality-checklist-database/). Reported health facility levels (ie, primary, secondary, and tertiary) was recorded directly from the program manual or source material.

### Data analysis

We conducted a content analysis of the qualitative interviewer notes from TRAction’s completed case studies across seven components: (1) means of quality assessment, (2) verification, (3) quality incentive formula, (4) quality assessment tool design, (5) impact of paying for quality, (6) challenges and (7) lessons learned was performed. We performed secondary comparative analysis using these same dimensions on the HFG the GDI case studies to reveal common themes related to better practices, challenges, and opportunities to improve quality of care in PBF. We grouped data on PBF program components into similarities and differences to highlight key thematic challenges and successes to using PBF to improve quality of care.

### Limitations

We relied on a small number of case studies that may not be representative of PBF programs in other contexts. All case studies were purposively selected, and although there is substantial heterogeneity in specific features and implementations of these cases, programs supported by the World Bank’s HRITF often have similar general design features [17]. Second, the case studies are limited to documentation and informants that were accessible to the study teams; these sources may not capture all details and experiences of the programs. Third, the case studies focus on a specific set of topics and may not cover other relevant issues and activities in PBF programs. Fourth, the quality checklist scores were often reported as an overall checklist score or by sub-indices [10-15], making it difficult to discern if specific indicators are performing well or poorly or if some are unattainable. In the case of Mozambique and DRC (PROSANI) no quality scores were ever publicly available but obtained with permissions. Zambia and Malawi’s PBF program provided no quality performance data. Vis-à-vis online RBF portals, quality scores from Senegal (2014-2015) [24], Nigeria (2015-2017) [25], and Kyrgyzstan (2016-2018) [26] were accessed (by sub-indices scores) for all facilities.

## RESULTS

**Figure 1:**
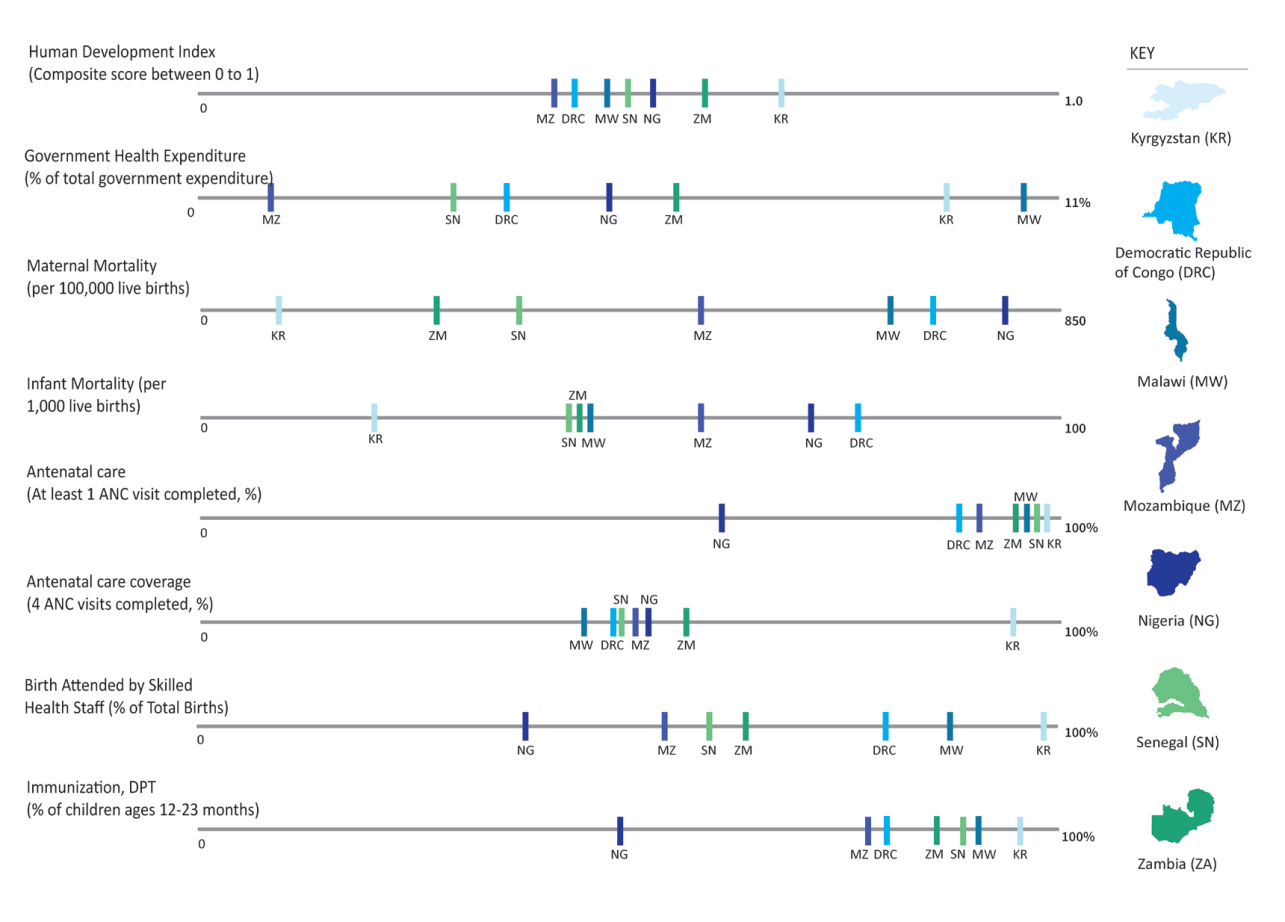
Cross country comparison of MCH context. Sources: All figures are from The World Bank Group. Retrieved from http://data.wordbank.org/ (2014) with the following exceptions: **Nigeria**, **Prenatal care visit (1 ANC) and skilled delivery** (Sources: The World Bank Group, 2013. Retrieved from http://data.wordbank.org/); **4 ANC visits completed** (Source: The World Health Organization, 2013. Retrieved from http://apps.who.int/gho/data/node.main.REPWOMEN39?lang=en); **Mozambique** **Prenatal care visit (1 ANC) and skilled delivery** (Sources: The World Bank Group, 2011. Retrieved from http://data.worldbank.org); **4 ANC visits completed** (Source: The World Health Organization, 2013. Retrieved from http://apps.who.int/gho/data/node.main.REPWOMEN39?lang=en); **Senegal**, 4 ANC visits completed (Source: The World Health Organization, 2012-2014. Retrieved from http://apps.who.int/gho/data/node.main.REPWOMEN39?lang=en); **Malawi, DRC, Zambia**, **4 ANC visits completed** (Source: The World Health Organization, 2013-2014. Retrieved from http://apps.who.int/gho/data/node.main.REPWOMEN39?lang=en); **Kyrgyzstan, 4 ANC visits completed** (Source: The World Health Organization, 2014. Retrieved from http://apps.who.int/gho/data/node.main.REPWOMEN39?lang=en).

Our findings are organized by similarities and differences observed across the seven PBF programs. In light of the country contexts, our analysis examined macro-economic figure and MCH outcomes (**Figure 1**). **Table 1** briefly describes the PBF programs while compares the key program components (described in further detail below).

**Table 1:** PBD program descriptions

Country	Description
**Nigeria**	The Nigerian Ministry of Health partnered with the World Bank and the Health Results Innovation Trust Fund to create a US$ 170 million performance based financing (PBF) scheme in 2011. The project was pre-piloted in one Local Government Authority (LGA) in each of three different states: Adamawa, Nasarawa, and Ondo and was gradually scaled up until January, 2015 when it reached coverage of 459 health centers and 26 hospitals. As of January 2015, the pilot covered approximately 50% of the LGAs in each of the three states. An additional financing of US$ 145 million for the NSHIP is being prepared. Of this financing, US$ 125 million is International Development Association (IDA), and US$ 20million will be from the Global Financing Facility (GFF). The operation in the north will focus on the States of Borno, Yobe, Gombe, Taraba and Bauchi states, with a particular attention for Borno and Yobe States in mid-2016.
**Mozambique**	The Elizabeth Glaser Pediatric AIDS Foundation (EGPAF) PBF pilot is the largest and longest running PBF program in the country. The pilot is financed by US President’s Emergency Plan for AIDS Relief (PEPFAR) through the United States Centers for Disease Control and Prevention (CDC) at 11 million USD for three years. The goals of the pilot are to accelerate the achievement of the Maternal and Child Health and HIV/AIDS-focused health outcomes. The PBF pilot was initiated in January 2011 in two provinces (Gaza and Nampula). As of 2015, the PBF program is continuing in the two pilot provinces in a total of 142 health facilities, 65 in Nampula and 77 in Gaza, equating to 31% and 57% population coverage respectively. This project is inactive as of 2016.
**Democratic Republic of Congo (DRC), PROSANI**	The PROSANI PBF program is a USAID-funded program co-implemented by the government of DRC and Management Sciences of Health (MSH). The PROSANI PBF implementation operated in four provinces, East Kasai, West Kasai, Katanga and South Kivu, with the goal of strengthening the health system and improving MCH, nutrition, and hygiene and sanitation. In 2013, the program included 118 health centers and seven general hospitals, which were all public health facilities. PROSANI was one of four PBF programs in DRC, as of 2015. Efforts to consolidate the PBF implementations were completed as of 2017, and the launch of a national PBF program was active as of 2018.
**Senegal**	The Senegal Ministry of Health began its own PBF pilot in 2012 after visiting the PBF program in Rwanda. The program is supported by USAID and the World Bank with the goal to motivate health workers, improve quality of care, improve health outcomes, and strengthen the capacity of district health teams. A pilot started in the Kolda and Kaffrine Districts of the Kolda and Kaffrine regions in 2012, and was expanded to cover all districts in these two regions in 2013, with an additional four regions in 2015. The Senegal PBF program has a rare form of pay for performance in that facilities and districts are paid against the achievement of coverage targets, which are negotiated in annual performance contracts. The quality score is then used to deflate the payment amount.
**Malawi**	The Results-Based Financing Initiative for Maternal and Neonatal Health (RBF4MNH) is supported by the German (KfW) and Norwegian governments, and uniquely intervenes on both the supply and the demand side. The project’s primary focus is to improve access to and quality of maternity, newborn and child health services. In April 2013, a pilot program across a cohort of 17 basic health facilities in four districts was initiated. The pilot was then expanded to cover the entirety of all four districts in 2014, including Mchinji, Dedza, and Ntcheu in the Central zone and Balaka district in the Southern zone).
**Kyrgyzstan**	The Kyrgyzstan PBF program, supported by the Health Results Innovation Trust Fund (HRITF) with a project budget of US$ 11 million USD (2014-2017), only includes secondary health facilities and focuses solely on quality of care. The Kyrgyz Health Results-Based Financing project comprises of two interlinked pilot interventions to reduce Kyrgyzstan’s persistently high maternal and neonatal death rates.
	The first pilot intervention consists of a randomized controlled trial implemented to test the feasibility and impact of a pay-for-quality performance-based financing (PBF) scheme at rayon (district) hospitals. The first pilot includes three arms, to which all district hospitals were randomly assigned: Arm 1 included 22 district hospitals receiving enhanced supervision to support quality improvement linked to performance-based payment based on hospital quality scores; Arm 2 included 21 rayon-level hospitals receiving enhanced supervision to support quality improvement only and no performance-based payments; and Arm 3 with 21 rayon-level hospitals receiving no interventions. Hospital quality is measured using a Balanced Score Card (BSC) approach. The second PBF intervention is soon to be piloted at the primary care level, providing PBF payments based on the quality and on the quantity of services delivered in four rayons.
**Zambia**	From 2008-2014, the Government of Zambia and the World Bank partnered on a project to design and implement a provider payment system that could accelerate the country’s reduction of under-five and maternal mortality in 11 districts (incrementally scaled). The pilot focused on rural areas for two reasons. First, maternal and child health status is lower in rural than urban areas. Second, 72% of the poor in Zambia live in rural areas, and the rural poverty rate is reportedly 80%. The Zambia RBF pays the providers for service provision and quality of high priority maternal and child health services. The project introduced a performance-based provider payment to motivate frontline health workers to work at full capacity and improve health service quality, as well as motivate District Medical Offices to fulfill critical supervisory and management functions.
	Starting in 2016, the Zambia Health Services Improvement Project (ZHSIP). The RBF component under the ZHSIP was officially launched and seeks to expand the RBF horizontally and vertically by the end of the project in June 2019 [2,3]. The ZHSIP program is funded by the HRITF US$ 15 million and IDS US$ 42 million for three years. Specific scale up goals include: Increased population coverage from 1.7 million to 4.4 million; Increase the number of districts from 11 to 39; and the number of health centers from 203 to 702; Introduce RBF in over 1500 community-based organizations.

PBF – performance-based financing

Four of the seven programs are supported by the World Bank, with the remaining three receiving support from USAID, KfW and the Centers for Disease Control and Prevention. Six programs target primary care, and five programs target multiple health facility levels (generally primary and tertiary care). All programs remain active as of this writing, with the exception of Mozambique and DRC PROSANI, which has been combined with other PBF programs in the country for national scale up.

### Similarities

#### Overview of quality checklists

The seven programs included 18 unique quality checklists, containing over 1400 quality of care indicators. On average, the checklists contained 116 indicators, ranging from 26 on the HIV-specific checklist for Mozambique to 228 on the tertiary quality checklist for Nigeria (**Table 2**). Interestingly, primary and tertiary level checklists did not differ considerably in the number of indicators (110 and 115 indicators on average, respectively). Informants reported time spent on quality checklists to consume a considerable percentage of verification efforts due to their length.

**Table 2:** Changes in the number of quality of care checklist indicators following checklist revisions

_Case study countries_													
**Comparative variables**	**Democratic Republic of Congo (PROSANI)**	**Malawi (RBF4MNH)**		**Kyrgyzstan**		**Mozambique**		**Nigeria**		**Senegal**		**Zambia**		
**Year (Year that is noted on the version of the checklist used)**	**(2014)**	**(2015)**		**(2014)**		**(2015)**		**(2014)**		**(2015)**		**(2012)**		
Funder	USAID	KfW, NORAD		World Bank		CDC		World Bank		World Bank		World Bank		
Geographic coverage of Program (number of facilities included)	80 health zones, 143 health centers	4 districts		National		2 provinces		3 states		3 districts		10 districts		
Payment type for quality*	Unconditional inflator	Unconditional inflator		Unconditional inflator (Arm 1)^†^		Inflator, threshold (≥60%)		Inflator, threshold (≥50%)		Unconditional deflator		Inflator, threshold (≥61%)		
Payment allocation per recipient, % of total payment (health facility, health providers)	40%, 60%	30%, 70%, 0%		40%, 25%, (35% flexible depending on facility preference)‡		60%, 40%, 0%		50%, 50%, 0%^d^		25%, 75%, 0%		75%, 25%, 0%§		
Number of quality indicators (by HS Level)	Primary: 143	Primary, secondary: 76		Secondary: 29		Primary, secondary (PCI): 179		Primary: 182		Primary: 72		Primary: 76	
	Tertiary: 158					Primary, Secondary, Tertiary (IMM; IMQ): 81, 26		Secondary: 228		Tertiary: 109			
Number of quantity Services (quantity indicators)	**15**		**11**		**N/A‖**		**21**		**20 MPA, 22 CPA**		**10**		**9**	
Verification frequency (quality)	Quarterly		Quarterly		Quarterly		Bi-annually		Quarterly		Quarterly		Quarterly	
Verifier (quality)	Regional govt. team & PROSANI team		Regional govt. team		Ex-ante through mixed team of consultants and Mandatory Health Insurance Fund staff, with peer hospital staff serving as observers		Regional govt. team + managing NGO		Ex-ante through district team for health centers, and through the Hospital management board for the hospitals		National & regional govt. team		Ex-ante, contracted hospitals (peer) and independent consultants	

CDC – Centers for Disease Control and Prevention, IMM – Maternal Health Checklist, IMQ – HIV/AIDS Checklists, KfW – German Development Bank, PCI – Prevention and Hygiene Checklist, USAID – United States Agency for International Development

*Payment Type: Conditional (threshold) deflator: Quality score deflates quantity payment continuously from 100% to minimum threshold. Payment is 0% if quality score is below the threshold. Continuous deflator (no threshold): Quality score deflates quantity payment continuously from 100% to 0% (no minimum threshold). Conditional inflator (Inflator, threshold): Quality score dictates amount of quality bonus received contingent upon achievement of a minimum quality score (threshold) required to receive any of the bonus. Unconditional inflator (no threshold): Quality bonus pool available; quality score dictates amount of quality bonus received.

†The first pilot intervention consists of a randomized controlled trial implemented to test the feasibility and impact of a pay-for-quality performance-based financing (PBF) scheme at rayon (district) hospitals. The first pilot includes three arms, to which all district hospitals were randomly assigned: Arm 1 included 22 district hospitals receiving enhanced supervision to support quality improvement linked to performance-based payment based on hospital quality scores; Arm 2 included 21 rayon-level hospitals receiving enhanced supervision to support quality improvement only and no performance-based payments; and Arm 3 with 21 rayon-level hospitals receiving no interventions. Hospital quality is measured using a Balanced Score Card (BSC) approach.

‡Currently, facilities can spend up to 25% of the bonus for staff incentives, in accordance with guidelines. Facilities are not allowed to use more than 40% of the payment for infrastructure improvements. Other than that, how the payment is spent is to a large degree up the facility.

§Note: in Nigeria and Zambia supervisors receive payment from a separate mechanism.

‖Uses a mixed global budget/output based financed budget (DRGs), and therefore is leveraging volume/quantity of patients.

#### Types of measures in the quality checklists

**Figure 2:**
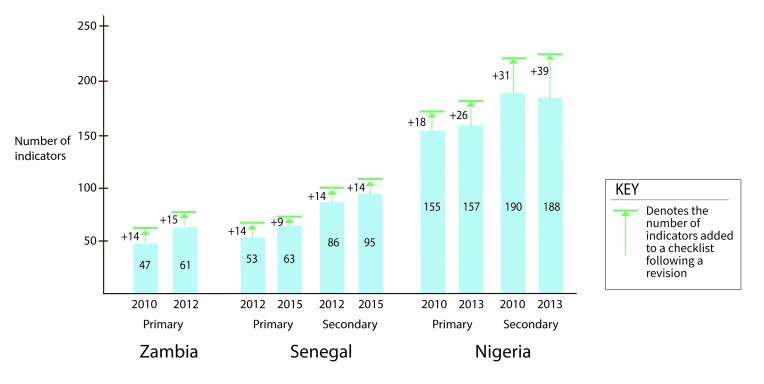
Changes in the number of quality of care checklist indicators following checklist revisions (4 schemes).

The quality checklists predominately measure structural components of quality (representing 80% of indicators on average, ranging from 38%-91%). Process indicators constituted an average of 20% across all checklists (ranging from 8.4% to 61.5%), with the majority measuring the correct application of care protocols for MCH services, including child immunization. The shares of structural and process indicators in programs for which we have multiple versions of revised checklists (Senegal, Nigeria, and Zambia), remained virtually unchanged post-revision, with the share of process indicators decreasing post-revision by 0.3% (of the overall proportion of the checklist), at the expense of additional structural indicators (**Figure 2)**.

Outcome indicators were mostly missing from these checklists. Only the tertiary care checklist from Kyrgyzstan contained a single outcome indicator, for patient satisfaction, which assesses the patient feedback survey of 10 patients, and is worth 10% of the checklist’s weight. Patient satisfaction was also measured in the DRC through four indicators on both the primary and tertiary checklists, worth 3.5% and 4% by weight, respectively. The tertiary list includes indicators that enforce the establishment of a patient satisfaction monitoring system, analysis of patient satisfaction data, and sharing of that analysis with health facility staff. In Senegal, Nigeria, and Zambia, patient satisfaction surveys are conducted as a part of community verification, but not factored into facility payment formulas or verified quality scores.

#### Clinical focus of the quality indicators

More than half of the checklist indicators for all programs focused on measuring the quality of facility management and planning (29%, on average by weight) and maternal health (22%), including antenatal, postnatal and delivery care. A few checklists had a specialty focus, specifically for Malaria prevention and treatment in Senegal, HIV/AIDS care and treatment in Mozambique (mixed level), and non-communicable diseases in Kyrgyzstan (tertiary level).

#### Positive quality performance trends

The aggregate quality scores for Nigeria, Mozambique, DRC, and Senegal, Kyrgyzstan demonstrate upward improvement in the median checklist performance over time (**Figure 2**). Aggregated program performance on quality checklists averaged 74% (in the last quarter, median also 74%) with an average increase of 16 percentage points in quality scores in eight quarters (comparing the checklist performance without any revisions). Across programs and quarters, primary and tertiary facilities achieved average scores of 63% and 57%, respectively. Introduction of a new or revised checklist and/or new verification training was associated with considerable variability in performance (not shown). For instance, in Nigeria, checklist revision was associated with initial decreased median performance, followed by recovery to previous achievement levels before plateauing at around 60%-70% in the quality checklist scores achieved over the ensuing three to four quarters.

#### Process of initial checklist design

Informants from all programs reported adapting existing quality tools to create PBF quality checklists. The main challenge for the programs was determining which aspects of existing tools were relevant for the context and forging consensus amongst disparate stakeholders. Over a year of debate in Kyrgyzstan, resulted in the technical working group adopting a balanced scorecard for tertiary facilities, based on an adaptation of an existing checklist for secondary health facilities (the 2009 version of the quality checklist from Rwanda). This process of adaption was nearly a three-year process due to political interruptions. The adapted tool was tested between January-June 2014 in one ‘pre-pilot' Rayon hospital and scaled after July 2014 to 44 tertiary facilities. Similarly, in Zambia, the ‘pre-pilot’ did not include a unique quality checklist. The pilot started with an adjusted (a modified and simpler) version drawn from the Rwandan health center quality checklist. Refinements to this quality checklist were informed by the pre-pilot results. Conversely in Mozambique, the initial quality checklist was the one already in use and designed by the Ministry of Health, however, as the program scaled up, quality checklists were developed by CDC (funder) and the program implementer (international NGO) for HIV and MCH-specific quality metrics using other non-PBF checklists as models.

#### Checklist revision

All programs revised their quality checklist in the last three years; however most were minimal. Informants stated that checklist reviews were often an annual part of program management, and the revision processes have been described as a “balancing act” between PBF expert guidance and existing quality tools used by country governments. In January 2014, Nigeria introduced a revised checklist, which included additional process-type indicators while maintaining the existing structural-type measures (process indicators increased from 11% to 27% in primary and 16% to 40% in secondary facilities, by weight). One rationale for frequent revisions according to informants is that facilities start mastering components on the original checklist and may hit the ceiling for improvement. The revision allows for further improvement in targeted services, thereby ensuring that facilities remain engaged and motivated to continually improve. Informants suggested a critical need for monitoring the implementation of quality checklists, including the length, frequency of verification, and the resources required to complete them.

#### Payment formula

Six of the seven programs use inflators for quality in their payment formulas (Payment type (inflator and deflator) is defined in the notes of **Table 2**. Three of the programs that inflate PBF payment is conditional (Mozambique, Nigeria, and Zambia), meaning the quality bonus is contingent upon achievement of a minimum quality score (threshold of 50% or 60%) to receive any of the quality bonus **(****Table 2**). Mozambique and Nigeria reported that some small rural facilities with poor existing infrastructure had difficulty achieving such thresholds of performance (50%-60%). For instance, in Mozambique, less than 15% of health facilities achieve the 60% threshold in any given quarter, meaning 85% of the facilities did not receive quality bonuses for any given quarter.

Senegal represents the sole use of a deflator scheme and can decrease the quantity of payment earned continuously from 100% to 0% based on the score a facility receives on the quality checklist. Zambia initially had the payment formula as a deflator; however, early program experience showed that individual-level bonuses earned were reportedly too low to increase worker motivation. After several consultations between the government and the funder, the quality deflator became an inflator, and eventually had conditionality of 61% achievement applied to it.

### Differences/challenges

#### Payment allocation and distribution

**Figure 3:**
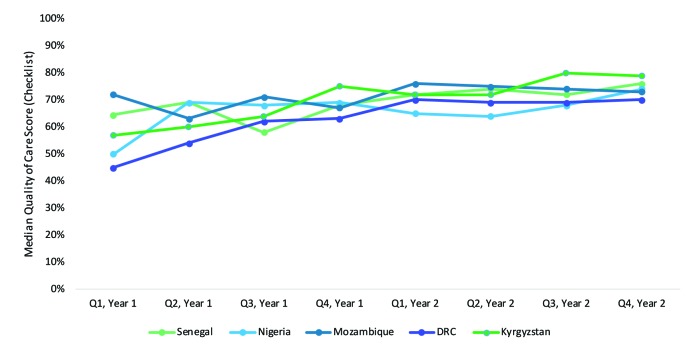
Median quality of care scores by quarter (data are presented for eight consecutive quarters between 2010 and 2015, during a period in which quality checklists did not undergo revision). Q1, Year 1 is the first quarter we have data recorded for. In many cases Q1 references one of the first quarters of quality checklist implementation. No revisions were introduced in any of the programs in the two year time period shown.

The PBF implementations exhibit high variability in bonus allocation between facility re-investment and health care provider (**Table 2**). Four programs’ allocation formulas favor staff incentives; the other three allocate a higher proportion (50%-75%) to facility re-investment. The programs in Mozambique, Malawi, and Zambia have separate bonus allocations to supervisors and district officials for good performance. No clear relationship exists between the countries that have higher proportion re-investment to facilities (50%-75%) and their performance on the quality checklists (**Figure 3**). In many PBF programs, facility re-investment is ‘investment units’ which are linked to business plans. These investment units are lumps sums and are transferred directly to health facility bank accounts (fiscal autonomy).

Financial data on quarterly or yearly quality allocations per geographic region or health facility is unavailable for all programs. Total payments across all health facilities is available for Nigeria from Q1 2015 to Q4 2017 totaling US$ 26.9 million both quantity and quality payments (average per quarter, US$ 2.24 million, median per quarter US$ 2.1 million) and Kyrgyzstan totaling US$ 6.3 million USD, for only quality, from Q2 2014 to Q4 2017 (average US$ 420 433 per quarter, median US$ 453 473 per quarter). For all other programs, the only financial data available at the time of this writing is the overall program budget across multiple years.

#### Level playing field for health facilities

Two programs provided health facilities with an initial investment in infrastructure and staff to ensure commencement of the PBF program with the required minimum standards for delivering quality care. In Zambia, implementation began with the provision of a package of reproductive health commodities and equipment to ensure that every facility could provide an acceptable level of quality for MCH services. A similar package was rolled out, including infrastructural upgrades, prior to the introduction of Malawi's PBF program.

#### Data and information transparency

Four programs have an online PBF data portal that reports quality checklist performance (Nigeria, Senegal, and Kyrgyzstan via RBF portals, and DRC via the PROSANI portal). Other programs do not have online portals and manage performance data through internal spreadsheets. While the online PBF portals have made great strides in enhancing data transparency, informants reported that these portals are rarely utilized at the health facility level, but rather by central level planning units, funders, and researchers. Much of the data available through the data portals is aggregated by region or by section of the quality checklist, rather than offering performance per indicator. In every case study, informants reported that health providers and community groups rarely receive their facility performance data and are not included in discussions on how to improve performance.

#### Verification processes

Across all programs, most indicators are measured via the checklist, whereby a verifier observes an indicator at a health facility and checks it off (79%), (eg, the presence of gloves, or presence of running water and soap). Typically, the programs only employ one or two supplemental means of assessment in addition to the checklist. Other methods include facility (5%) and patient register reviews (7%), direct observation of patient-provider interactions (8%), and staff assessments (<1%). There is also ex-post verification, which is the random checking of performance values after the payment is completed. Typically, there are penalties for discrepancies larger than 10%. This verification method is being deployed in Nigeria and Kyrgyzstan.

## DISCUSSION

Our findings indicate considerable heterogeneity in the design and implementation of quality of care components in select PBF programs. Payment allocations (distribution), data transparency, and measuring quality appear to differ across schemes, while the process of checklist design and revision, positive performance gains, payment formulas, and indicator typology are shared features. While the similarities and difference amongst program design and implementation serve as an important contribution to helping improve and re-design programs, the core finding of this paper is that PBF program information is burdensome to access, understand, and leverage for learning and comparison.

The current evidence gaps in understanding how PBF programs work mechanically and on-a-ground-level results directly from the dearth of information that is publicly available. For all of the case studies, program documents were obtained only through direct request and follow-up with the funder. This documentation differed significantly across programs and funders, particularly when comparing manuals, which are intended to have a shared format. Routine implementation documentation and noted discrepancies between design and execution are completely absent, even internally for many programs. Evaluations of the programs are spaced by years and take significant time to be published and released. Moreover, the formal evaluations provide little information about the actual implementation, with the focus being on impact.

Although great strides to increase data transparency has improved access to PBF data for HRITF-supported programs, few offer any analysis of this data, and in even fewer instances is this data used by the beneficiaries and verifiers, where it could be leveraged to strength programs.

A final glaring gap in the PBF information sphere is financial data. With regards to the topic of our analysis, the payments for quality remain elusive. While some programs, notably Nigeria and Kyrgyzstan, publish aggregate quarterly payment information on the public portal, how the payments are calculated, distributed amongst PBF facilities, and most importantly, how the money is spent overall and directly tied to the improvement in quality is measured on the checklist is critical to understanding whether the payment side of PBF has any effect on quality.

While the amount paid for quality is not available, our analysis provides insights into the payment formulas. PBF programs reviewed here favor incorporating quality as an inflator to quantity bonuses. However, the allocation (distribution) of payments and threshold of performance on the checklists differ. The experience of Senegal concerning the accountability and enforcement of the deflation approach was positive, however, most informants reported that inflators are more aligned with work culture and preference of health facility staff and managers. Payment thresholds may favor well-performing or well-equipped facilities. Conversely, this may demotivate smaller, lower-performing facilities that are unlikely to meet the threshold (as the case of Mozambique suggests), as well as higher-performing facilities who have no incentive to improve beyond just above the threshold. Some programs address this issue by graduating payment thresholds so that greater performance continues to increase payment incrementally. Periodic checklist revisions also help to address the problem of complacency.

Another shared challenge is the length and overt focus on structural quality components. The focus of the checklists on structural attributes is likely an acknowledgment that, in many LMICs, health facilities do not achieve a minimal level of infrastructure, supplies, and cleanliness. However, the process and outcome aspects of quality are increasingly recognized as important [27,28] and efforts are afoot to incorporate more of these measures in PBF quality checklists – while recognizing the potentially higher costs of routinely collecting and verifying such data. New approaches to measurement and verification – such as tablet-based verification and risk-based verification [29]–could lower the costs of using such measures in PBF programs. Similarly, there may be substantial scope to learn from quality improvement and monitoring programs outside of PBF, including from the respectful maternity care [30] and broader experience of care [31] movements.

Our finding that quality checklists are lengthy is echoed by a recent study assessing 68 quality of care checklists which found that checklists contained on average 126 indicators, ranging from 26 to 220 [23]. Key informants in our study suggested a critical need for monitoring specific aspects of the implementation of quality checklists. Facilities and supervisory teams also face an associated burden to prepare and conduct verification activities [29,32,33]. Risk-based verification based on historical data and predictive algorithms could improve on commonly used random sampling methods and has been piloted in Benin and Zimbabwe.

Enhanced role of patient feedback would be a positive shift in assessing quality, also to raise the awareness and responsiveness of providers to patient experiences. Moreover, there is a need for innovation to collect these kinds of measures at low cost. Enhanced engagement of the community in knowing and understanding facility quality performance could support a more participatory process to improve quality of care in PBF programs. In Kyrgyzstan, although it requires the facility to report at least patient feedback surveys, it does not specify a minimum score reporting minimum satisfaction, the payment is made for the completion.

Finally, this analysis points to the need to work more closely with continuous quality improvement (QI) and quality assurance (QA) programs when designing PBF programs. We know that other countries have concurrent QI/QA projects in the same facilities as PBF. Indeed, at least some of our sample countries – Mozambique, Nigeria, Malawi – have QI/QA efforts under way in the same provinces or districts, if not in the same facilities. The challenge ahead lies in the opportunity to creatively test and combine efforts between PBF and QI/QA processes to reduce redundancy and improve quality of care using multiple approaches.

## CONCLUSIONS

PBF holds appeal as a tool to improve the quality of care in health facilities and to focus decision-makers' attention on current quality shortfalls as well as toward performance-based payments. The growing operational experiences with PBF programs in different settings offer opportunities to learn from best practices and mistakes, improve ongoing and future programs, and inform research to alleviate challenges in current programs, such as how to shift toward process and outcomes quality while containing the administrative burden of reporting and verifying performance.
